# Simulation analysis for tumor radiotherapy based on three‐component mathematical models

**DOI:** 10.1002/acm2.12516

**Published:** 2019-03-12

**Authors:** Wen‐song Hong, Gang‐qing Zhang

**Affiliations:** ^1^ Radiotherapy Department of Guangdong Second Provincial General Hospital Guangzhou China

**Keywords:** computer simulation, three‐component mathematical model, tumor radiotherapy

## Abstract

**Objective:**

To setup a three‐component tumor growth mathematical model and discuss its basic application in tumor fractional radiotherapy with computer simulation.

**Method:**

First, our three‐component tumor growth model extended from the classical Gompertz tumor model was formulated and applied to a fractional radiotherapy with a series of proper parameters. With the computer simulation of our model, the impact of some parameters such as fractional dose, amount of quiescent tumor cells, and *α*/*β* value to the effect of radiotherapy was also analyzed, respectively.

**Results:**

With several optimal technologies, the model could run stably and output a series of convergent results. The simulation results showed that the fractional radiotherapy dose could impact the effect of radiotherapy significantly, while the amount of quiescent tumor cells and *α*/*β* value did that to a certain extent.

**Conclusions:**

Supported with some proper parameters, our model can simulate and analyze the tumor radiotherapy program as well as give some theoretical instruction to radiotherapy personalized optimization.

## 1 INTRODUCTION

Cancer may be the first terrible enemy of our mankind. Although there are a lot of exciting progresses in medical fields to help us for more healthy life, some cancers are still keeping threatening to the world, for example, lung cancer.^1^, ^2^ Nowadays, there are many kinds of techniques for cancer treatment, among which, surgery, radiotherapy, and chemotherapy may be the dominant ones.

The metabolic process of cancer is so complicate that its mechanism is still not revealed completely until now. Researchers try their best to develop many models for clinical treatment of cancer including mathematical models, which were proposed in the early 1900s and deepened in this century with the development of computer.^3^, ^4^ In these models, the features of tumor growth have been deduced into some basic mathematical theories such as signal processing, image analysis, and stochastic field theory, then, all the models were formulated mathematically according to the different theories and fitted with huge experimental or clinical data for tumor growth prediction and effective evaluation of tumor treatment.^5^

An ordinary differential equation (ODE), a classic applied mathematical analysis tool, has been used widely for tumor growth analysis and simulation.^6^, ^7^, ^8^ Because of its advantages of simplicity and good convergency, ODE can be easily handled by software with micro computer, so, with rapid development of computer, there have been many improvements in clinical research of the models based on ODE, such as Gompertz model (GM), power law model, and generalized logistic model.^9^, ^10^, ^11^

In this paper, we construct a three‐component (3‐C) tumor growth model for simulation the tumor metabolism, and we also introduce the GM for tumor growth process as well as linear–quadratic (L–Q) model for tumor radiotherapy. Then, our model is used for simulation of tumor fractional radiotherapy to discuss some proper parameters for radiotherapy optimization.

## 2 MODELS AND METHODS

### 2.A Gompertz model

The GM was proposed for tumor growth process in 1925 by Benjamin Gompertz, a British mathematician. It is given by 11

(1) $$\frac{\mathrm{dT}}{\mathrm{dt}}=aT-bTln\left(T\right)$$

where *T* is the tumor volume (cm^3^), *t* is the time, *ln*() is the natural logarithm, and *a* and *b* are constants. The model can converge to a constant *K* = *T*_0_ *e* ^*a*/*b*^, where *T*_0_ is the initial tumor volume and e is the natural constant. Then, *K* means the tumor capacity.

### 2.B Three‐component model for tumor growth

**Figure 1:**
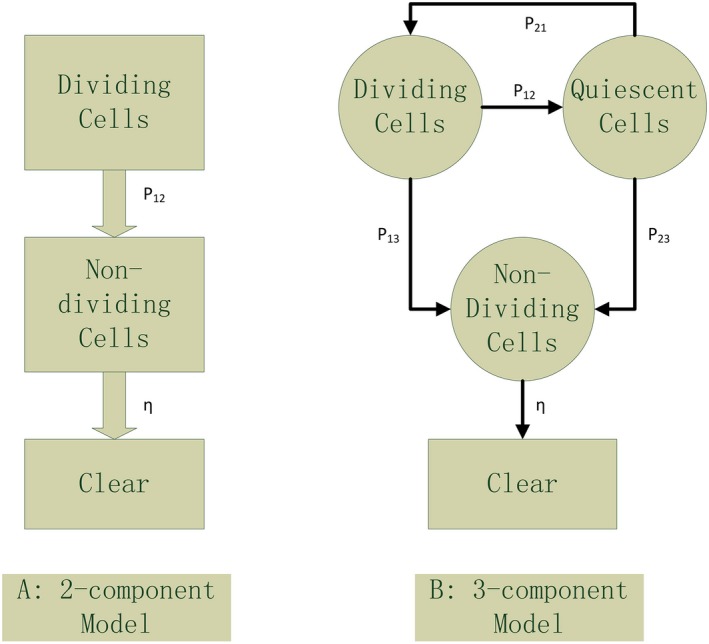
Comparison of two‐component model and three‐component model.

The essence of malignant tumor growth is the unordered and rapid division of tumor cells. Generally, in many papers, to simplify the analysis model, the tumor cells are divided into two groups: dividing cells and nondividing cells. The tumor growth relies mainly on the dividing cells, and the nondividing cells will dead naturally and be cleared successively by the body. This model is called two‐component Model (2‐C Model) [Fig. 1(a)].^12^, ^13^ Obviously, the quiescent tumor cells are neglected in this model. In fact, the quiescent cell may play an important role in tumor growth. Under some conditions, the quiescent cells may change to the dividing cells to impact the tumor growth. For more actual analysis, a 3‐C model is proposed here [Fig. 1(b)]. In our model, there are three kinds of tumor cells: dividing cells, nondividing cells, and quiescent cells. The quiescent cells may change to dividing cells or nondividing cells at certain probability. Its ODE model is given by

(2) $$\left\{\begin{array}{cc} \frac{dT_{A}}{\mathrm{dT}} & =aT_{A}-bT_{A}ln\left(T_{A}\right)-\left(P_{12}+P_{13}\right)T_{A}+P_{21}T_{Q}\\ \frac{dT_{Q}}{\mathrm{dt}} & =P_{12}T_{A}-\left(P_{21}+P_{23}\right)T_{Q}\\ \frac{dT_{D}}{\mathrm{dt}} & =P_{13}T_{A}+P_{23}T_{Q}-\eta T_{D} \end{array}\right.$$

where *P*_ij_ is the change probability from state *i* to state *j*,* η* is the clear rate, and *T*_A_, *T*_Q_,and *T*_D_ are dividing cells, quiescent cells, and nondividing cells, respectively.

### 2.C Radiotherapy model

In radiotherapy, with the different characteristic, the interaction between radiation rays and tumor cell is very different. For x ray or *γ* ray, the L–Q model is the most popular and widely used.^14^, ^15^ Its ODE formulation is:

(3) $$\frac{\mathrm{dT}}{\mathrm{dt}}=-\left(\alpha D+2\beta D^{2}\right)T$$

where *T* is the tumor volume, *D* is the radiation dose, and *α* and *β* are the coefficient of linear and quadratic item, respectively. Normally, the radiation sensitivity of the tumor cells can be described with *α*/*β*. As we know, the tumor cells in different state will have different radiation sensitivity. So, in this paper, it is assumed the radiation rays only act on the dividing and quiescent cells with different sensitivity. The ODE model is:

(4) $$\left\{\begin{array}{cc} \frac{dT_{A}}{\mathrm{dT}} & =aT_{A}-bT_{A}ln\left(T_{A}\right)-\left(P_{12}+P_{13}\right)T_{A}+P_{12}T_{Q}-\left(\alpha_{1}D+2\beta_{1}D^{2}\right)T_{A}\\ \frac{dT_{Q}}{\mathrm{dt}} & =P_{12}T_{A}-\left(P_{21}+P_{23}\right)T_{Q}-\left(\alpha_{2}D+2\beta_{2}D^{2}\right)T_{Q}\\ \frac{dT_{D}}{\mathrm{dt}} & =P_{13}T_{A}+P_{23}T_{Q}-\eta T_{D} \end{array}\right.$$

where *α*_1_, *β*_1_ and *α*_2_, *β*_2_ are the radiation sensitivity of dividing cells and quiescent cells, respectively.

Now, fractional radiotherapy is the dominant plan in routine radiotherapy. It is necessary to consider the tumor cell proliferation and the change in quiescent cells during the gap between two fractions. Then, the ODE model is unfit for simulating the process. Here, we propose a piecewise integration model for fractional radiotherapy simulation:

(5) $$\begin{array}{cc} T_{A}=\; & \sum_{i=1}^{N}(\int_{t0}^{t*}-\left(\alpha_{1}D_{i}+2\beta_{1}D_{i}^{2}\right)T_{\mathrm{Ai}}dt\\ & +\int_{t0}^{\mathrm{td}}(aT_{\mathrm{Ai}}-bT_{\mathrm{Ai}}\ln\left(T_{\mathrm{Ai}}\right)-\left(P_{12}+P_{13}\right)T_{\mathrm{Ai}}\\ & +P_{21}T_{\mathrm{Qi}})dt) \end{array}$$

where *N* is the total radiotherapy fractions, (*t*0, t*) is the radiation time, (*t*0, *td*) is the time between to fractions, *D*_i_ is the radiation dose of the fraction *i*, and *T*_Ai_ and *T*_Qi_ are the volume of *T*_A_ and *T*_Q_ at fraction *i*, respectively. We can also formulate the model of *T*_Q_ in the same way.

### 2.D Numerical simulation

A computer software is developed for simulating the model. The programming language is Matlab R 2016a (Mathworks corporation, Natick, MA, USA). Parts of the model parameters are list in Table 1.^5^

**Table 1:** Partial parameters for the model

GM	Three‐component model
*a* = 0.56, *b* = 0.0719	*a* = 0.653, *b* = 0.0719 *P*_12_ = 0.1, *P*_21_ = 0.1, *P*_13_ = 0.05, *P*_23_ = 0.05, *η* = 0.2
*a* = 0.742, *b* = 0.0792	*a* = 0.837, *b* = 0.081 *P*_12_ = 0.1, *P*_21_ = 0.1, *P*_13_ = 0.05, *P*_23_ = 0.05, *η* = 0.2

## 3 RESULTS AND ANALYSIS

### 3.A Simulation and analysis for tumor growth model

**Figure 2:**
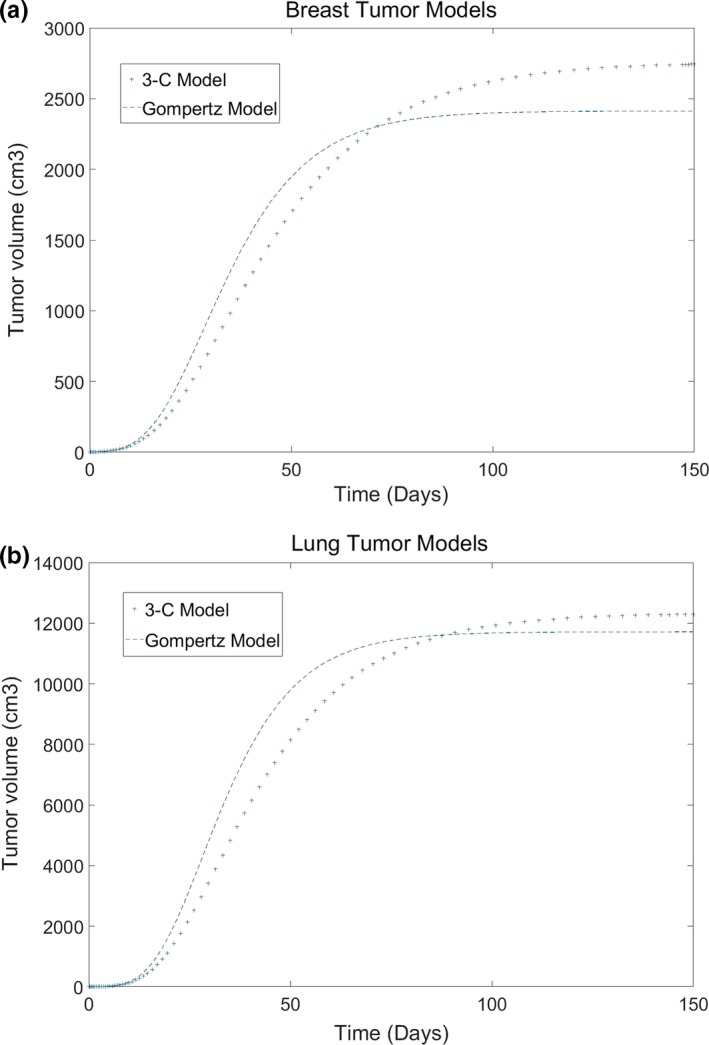
The difference of tumor growth between GM and 3‐C model (a: Breast cancer, b: Lung cancer).

During the early stage, the tumor cells increase exponentially. While reaching certain volume, the growing trend is slow down (Fig. 2). It shows that both the models can return the similar results. Because of the capacity constraint of GM, its curve runs like a horizontal line finally and shows a hardly growing tumor volume, while in the 3‐C model, the curve still rises slowly at the end. The growing trend of 3‐C model is controlled by the probability from quiescent cells to dividing cells and nondividing cells.

### 3.B Impact of fractional dose to radiotherapy result

**Figure 3:**
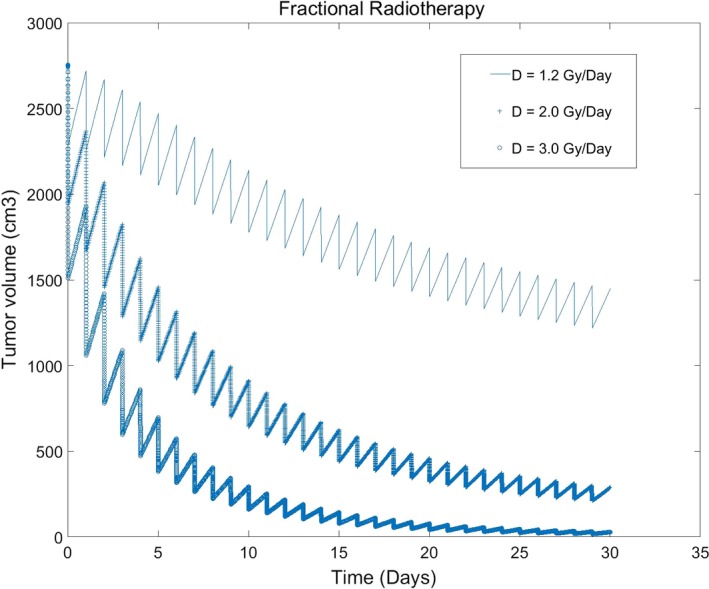
Impact of different fractional doses to tumor volume (fractional dose: 1.2, 2.0, 3.0 Gy/Day, 30 times; *α* ^1^/*β* ^1^ = 10; *α* ^2^/*β* ^2^ = 6.6).

Generally, larger the fractional dose is, better the tumor control is, and rapider the convergency of the model is. In Fig. 3, when the fractional dose is 1.2 Gy, total treatment of 30 times (cumulative dose = 36Gy) cannot reach the control result. While the fractional dose is 3.0 Gy, total treatment of 12 times (cumulative dose = 36 Gy) can control the tumor volume under 5% of its initial volume. Of cause, higher fractional dose will do more harmful to the surrounding normal tissues. For tumor radiotherapy optimization, it is necessary to consider the radiation models for normal tissues as well as the ones for the tumor.

### 3.C Impact of quiescent cell volume to radiotherapy result

**Figure 4:**
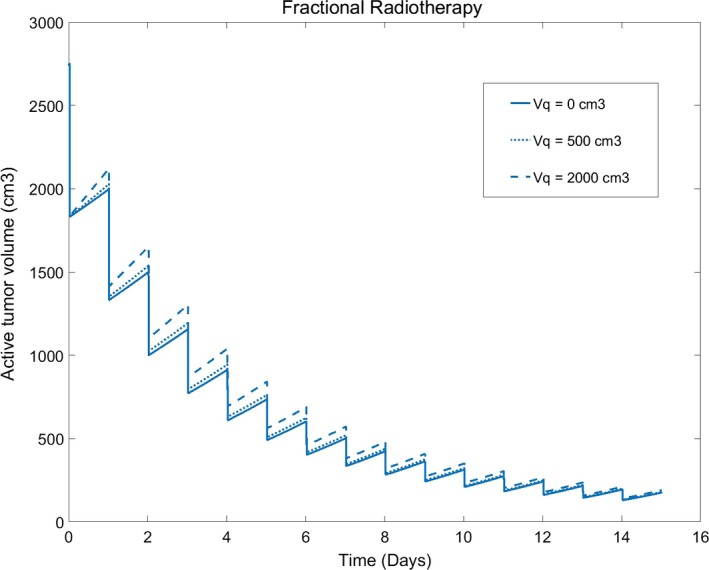
Impact of quiescent cells volume to the tumor volume (quiescent cells Vq: 0, 500, 200 cm^3^; fractional dose: 2.0 Gy/Day, 15 times; *α* ^1^/*β* ^1^ = 4.5; *α* ^2^/*β* ^2^ = 10).

In our 3‐C model of tumor growth, the impact of quiescent cells to the tumor growth can be seen. In Fig. 4, it can be concluded that the initial volume of quiescent cells impacts the process of radiotherapy first, then, as soon as smaller the volume of quiescent cells is, the weaker the impact is.

### 3.D Impact of the value of *α*/*β* to radiotherapy result

Obviously, we can read from the model that the impact facts of quiescent cells to tumor radiotherapy include the initial volume as well as the probability from quiescent cells to other cells and the value of α/β, the parameters of radiation sensitivity. All the parameters may be so important for the model application to clinical radiotherapy.

**Figure 5:**
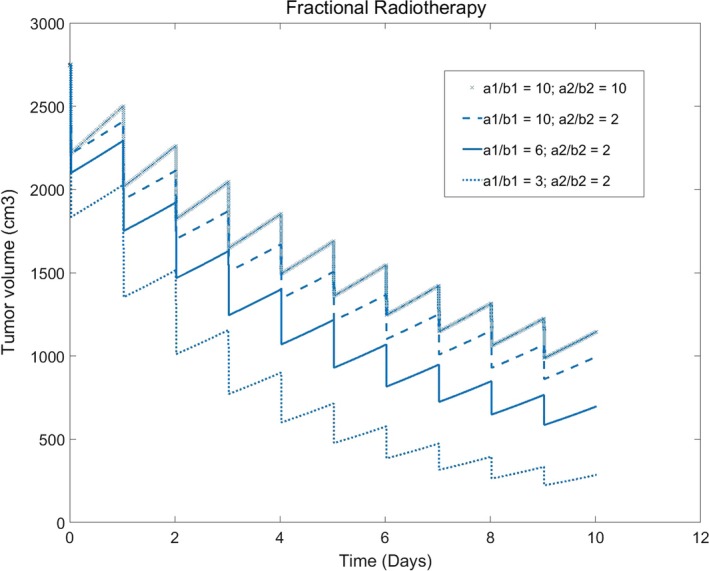
Impact of radiation sensibility to the tumor volume (fractional dose: 3.0 Gy/Day, 10 times)

*α*/*β* is the indicator of the radiation sensibility of tumor cells. Generally speaking, larger the ratio of *α*/*β* is, the linear action of L–Q model is more significant than the quadratic action. In the same conditions, larger the ratio of *α*/*β* is, flatter the curve of the tumor control is, and more fractional times or dose are needed (Fig. 5). In our model, because of the action of quiescent cells, the simulation results are also impacted by *α*_2_/*β*_2_, the radiation sensitivity of quiescent cells. We can analyze from the model and Fig. 5 that larger the ratio of *α*_2_/*β*_2_ is, poorer the radiotherapy effect is.

## 4 DISCUSSIONS AND CONCLUSION

In the field of radiotherapy model, there may be two branches. One is about general model of tumor growth, and another is about the interaction model of tumor cells and radiation particles. In general tumor model, GM may be one of the most classic mathematical model. This kind of models describes the intricate and comprehensive biological activities with some compact formulations and opens a new window for tumor basic research. Because of the complexity of biomedical activities and the limitation of the research conditions, this model can only be used for basic analysis of tumor features. As we know, all mathematical models cannot be useful in clinic until serials of proper quantized model parameters are introduced. To find the solution, based on the classic models, many researchers try their best to propose some achievable models for guiding the clinical research according the tumor features.^16^, ^17^, ^18^, ^19^ For example, Costa et al.^20^ formulated a tumor growth mathematical model with some real parameters refined *in vitro*, and Roberto et al.^21^ published a paper in 2015 to reveal the effect of obesity on cancer growth and on the immune system response using mathematical model. Their model discussed the quantitative relationship between obesity and tumor in order to find a valuable diet planning for clinical tumor prevention and treatment. In other papers, mathematical model was combined with the technique of medical image processing for evaluation and prediction of tumor multidisciplinary treatment.^22^ Although there has not been a comprehensive mathematical model for clinical application until now, some positive improvements have been achieved in serials of fields.^23^, ^24^ It is assured that the tumor mathematical models will reach an excellent level with the rapid development of computers in the near future.^25^

The research of the interaction model between radiation particles and tumor cells has started since 1960s and formatted the widely used L–Q model with constantly improvement.^14^, ^15^, ^26^ In current papers, there are many exciting results in radiotherapy effect using the general mathematical model combined with the L–Q Model.^27^, ^28^, ^29^ We find that the model in the most of the papers is the 2‐C one and the quiescent tumor cells are not considered. In this paper, we attempt to propose a 3‐C tumor model for analysis of the quiescent cells effect. The simulation gives us positive evidence that the initial volume of quiescent cells and the radiation sensitivity coefficient can impact radiotherapy effect. That is to say, with more accurate model and real model parameters, the 3‐C tumor model can give a hand in the clinical field of tumor radiotherapy optimization. Some papers show us that radiation sensitivity and fractional dose are related to many human biological indexes, for example, gene and protein.^30^, ^31^, ^32^ With the studies, it is possible to quantify the association between the indexes and our model parameters, and some patient‐specific parameters can be extracted and fitted with the real biomedical data. That may be the next step of our research.

## CONFLICT OF INTEREST

The authors declare no conflict of interest

## References

[1] Jemal A , Bray F , Center MM , et al. Global cancer statistics. CA Cancer J Clin. 2011;61:69–90. 21296855 10.3322/caac.20107

[2] Ferlay J , Soerjomataram I , Dikshit R , et al. Cancer incidence and mortality worldwide: sources, methods and major patterns in GLOBOCAN 2012. Int J Cancer. 2015;136:E359–E386. 25220842 10.1002/ijc.29210

[3] Bratus AS , Fimmel E , Kovalenko SY . On assessing quality of therapy in non‐linear distributed mathematical models for brain tumor growth dynamics. Math Biosci. 2014;248:88–96. 24384228 10.1016/j.mbs.2013.12.007

[4] Nazila B , Lotfi MM . A multi‐objective multi‐drug model for cancer chemotherapy treatment planning: a cost‐effective approach to designing clinical trials. Comput Chem Eng. 2016;87:226–233.

[5] Sébastien B , Clare L , Afshin B , et al. Classical Mathematical Models for Description and Prediction of Experimental Tumor Growth [OL]. https://arxiv.org/pdf/1406.1446. 10.1371/journal.pcbi.1003800 PMC4148196 25167199

[6] MartõÂnez‐RincoÂn RO , Rivera‐PeÂrez C , Diambra L , Noriega FG . Modeling the flux of metabolites in the juvenile hormone biosynthesis pathway using generalized additive models and ordinary differential equations. PLoS ONE. 2017;12:e0171516. 28158248 10.1371/journal.pone.0171516 PMC5291429

[7] Laurent‐Puig P , Manceau G , Boige V , Blons H . Prediction of response to anticancer treatment as simple as the resolution of ordinary differential equations? Gut. 2012;61:637–638. 22203374 10.1136/gutjnl-2011-301767

[8] Martin B , Mikael S , Martin A , et al. Investigations of a compartmental model for leucine kinetics using non‐linear mixed effects models with ordinary and stochastic differential equations. Math Med Biol. 2012;29:361–384. 21965323 10.1093/imammb/dqr021

[9] Castorina P , Diesboeck TS , Gabriele P , et al. Growth laws in cancer: implications for radiotherapy. Radiat Res. 2007;168:349–356. 17705631 10.1667/RR0787.1

[10] West GB , Brown JH , Enquist BJ . A general model for ontogenetic growth. Nature. 2001;413:628–631. 11675785 10.1038/35098076

[11] José SD . Gompertz model: resolution and analysis for tumors. J Math Modell Appl. 2012;1:70–77.

[12] Yoichi W , Erik LD , Kevin ZL , et al. A mathematical model of tumor growth and its response to single irradiation. Theor Biol Med Model. 2016;13:6. 26921069 10.1186/s12976-016-0032-7 PMC4769590

[13] Chvetsov AV , Dong L , Palta JR , et al. Tumor‐volume simulation during radiotherapy for head‐and‐neck cancer using a four‐level cell population model. Int J Radiat Oncol Biol Phys. 2009;75:595–602. 19596173 10.1016/j.ijrobp.2009.04.007

[14] Matthias G , Rainer JK , Michael A , et al. Applicability of the linear‐quadratic formalism for modeling local tumor control probability in high dose per fraction stereotactic body radiotherapy for early stage non‐small cell lung cancer. Radiother Oncol. 2013;109:13–20. 24183066 10.1016/j.radonc.2013.09.005

[15] Masahiro M , Seishin T , Hiroyuki D , et al. A mathematical study to select fractionation regimen based on physical dose distribution and the linear quadratic model. Int J Radiat Oncol Biol Phys. 2012;84:829–833. 22417807 10.1016/j.ijrobp.2012.01.004

[16] Colin P , Hamid M , Mohammad K . A microscale mathematical model for metabolic symbiosis: investigating the effects of metabolic inhibition on ATP turnover in tumors. J Theor Biol. 2015;366:103–114. 25433213 10.1016/j.jtbi.2014.11.016

[17] Nitish P , Feba SS , Wayne C , et al. A three dimensional micropatterned tumor model for breast cancer cell migration studies. Biomaterials. 2016;81:72–83. 26724455 10.1016/j.biomaterials.2015.11.039

[18] Hong W . Simulation analysis of tumor combination therapy based on mathematical models. China Dig Med. 2016;11:33–36.

[19] Kim Y , Jeon H , Othmer H . The role of the tumor microenvironment in Glioblastoma: a mathematical model. IEEE Trans Biomed Eng. 2017;64:519–527. 27959794 10.1109/TBME.2016.2637828 PMC5384419

[20] Costa FH , Campos M , Aiéllo OE , et al. Basic ingredients for mathematical modeling of tumor growth in vitro: cooperative effects and search for space. J Theor Biol. 2013;337:24–29. 23954328 10.1016/j.jtbi.2013.07.030

[21] Roberto AK , Sandra ED , Chen‐Charpentier BM . A mathematical model for the effect of obesity on cancer growth and on the immune system response. Appl Math Model. 2015;40:4908–4920.

[22] Yoshiharu O , Hisanobu K , Yasuko F , et al. Dynamic contrast‐enhanced perfusion area detector CT for non‐small cell lung cancer patients: influence of mathematical models on early prediction capabilities for treatment response and recurrence after chemoradiotherapy. Eur J Radiol. 2016;85:176–186. 26724663 10.1016/j.ejrad.2015.11.009

[23] Steuperaert M , Falvo DL , Debbaut C , et al. Mathematical modeling of intraperitoneal drug delivery: simulation of drug distribution in a single tumor nodule. Drug Deliv. 2017;24:491–501. 28181817 10.1080/10717544.2016.1269848 PMC8240979

[24] Bonilla LL , Capasso V , Alvaro M , et al. On the mathematical modeling of tumor‐induced angiogenesis. Math Biosci Eng. 2017;14:45–66. 27879119 10.3934/mbe.2017004

[25] Ella H , Eiman TA , Neveen IG . Computational intelligence techniques in bioinformatics. Comput Biol Chem. 2013;47:37–47. 23891719 10.1016/j.compbiolchem.2013.04.007

[26] Francisco CG , Consuelo PV , María HC , et al. Assessment of radiobiological metrics applied to patient‐specific QA process of VMAT prostate treatments. J Appl Clin Med Phys. 2016;17:341–367. 10.1120/jacmp.v17i2.5783 PMC7711539 27074458

[27] Jacqueline AE , Jos T , Martine K , et al. Prediction model to predict critical weight loss in patients with head and neck cancer during (chemo)radiotherapy. Oral Oncol. 2016;52:91–96. 26564309 10.1016/j.oraloncology.2015.10.021

[28] Anja B , Udo S , Gero W . Simulation of metastatic progression using computer model including chemotherapy and radiation therapy. J Biomed Inform. 2015;57:74–87. 26190266 10.1016/j.jbi.2015.07.011

[29] Mohammed AS . Intrinsic radio‐sensitivity of tumors to low let radiations: a mathematical model in LQ formalism. Med Hypotheses. 2013;81:1041–1045. 24182870 10.1016/j.mehy.2013.09.031

[30] Jacob GS , Anders B , Michael JS , et al. A genome‐based model for adjusting radiotherapy dose (GARD): a retrospective, cohort‐based study. Lancet Oncol. 2017;18:202–211. 27993569 10.1016/S1470-2045(16)30648-9 PMC7771305

[31] Zhi H , Ge H , Anguraj S , et al. The expression level of HJURP has an independent prognostic impact and predicts the sensitivity to radiotherapy in breast cancer. Breast Cancer Res. 2010;12:R18–R32. 20211017 10.1186/bcr2487 PMC2879562

[32] Navita S , John Y , Frances D , et al. The relationship between homologous recombination repair and the sensitivity of human epidermis to the size of daily doses over a 5‐week course of breast radiotherapy. Clin Cancer Res. 2012;18:5479–5488. 22855580 10.1158/1078-0432.CCR-10-3297

